# Extended Weekly Contributions in London

**Published:** 1920-11-20

**Authors:** 


### The Hospital

The Workers' Newspaper of Administrative Medicine and Institutional Life, Administration, National Insurance and Health.

No. 1798, Vol. LXIX. SATURDAY, NOVEMBER 20, 1920. PRICE TWOPENCE

### EXTENDED WEEKLY CONTRIBUTIONS IN LONDON.

There really seems a chance that a system of weekly contributions to hospitals will develop generally in London. We have advocated so often efforts to extend these contributions, both within and without the sphere of the Metropolitan Hospital Saturday Fund, that we welcome assistance to the same end from every quarter. Recently a correspondent in our own columns urged that the heads of other great firms with numerous branches should encourage these to follow the example set by Messrs. W. H. Smith & Son's staff, and start a weekly contribution of twopence. In this case, as we have already reported, King's College Hospital is the beneficiary. By a fortunate coincidence the action of this firm inspired not only our correspondent but the Editor of the *Daily Express*, who advocated its general adoption, and in support of its idea sought the opinion of various hospital men, including Mr. Inman of the Hospital Saturday Fund. Mr. Inman put the case in a nutshell when he replied: "There is room for a new scheme beside our own, though the machinery of the fund is available for the allotment of all contributions." Lord Hambleden, the Chairman of King's College Hospital, declared, with his own firm in mind no doubt, that the plan could be generally adopted; and that, were it adopted, the hospitals, he thought, could offer to the large firms whose staffs subscribed free out-patient treatment and perhaps in-patient treatment at reduced rates. The value of this inducement to large firms and to their staffs, Lord Hambleden is in an excellent position to judge. This being so, we venture to suggest that no one is so well fitted as he to become the missionary of the plan. With the experience of Messrs. W. H. Smith & Son behind him, and his position as Chairman of King's College Hospital, can he not interest other large employers in the scheme? The names of Sir Jesse Boot, Mr. Gordon Selfridge, and Sir Woodman Burbidge have already been mentioned in these columns, but we have lacked hitherto a large employer, with hospital experience, whose staff has put the plan into practice.

These combined advantages have now fallen to Lord Hambleden, and, since he must be as anxious as any man to secure further weekly contributions for his own hospital, we hope that he will not lose the present opportunity, when the idea is in the air, to press the scheme upon those best able to encourage it. The *Daily Express* has done great service by the publicity which it has given to the action of Messrs. W. H. Smith's employees; and we were glad to see signs that the same paper is collecting instances of other London firms, like Messrs. Bryant & May, where hospital collections are in force. We cannot urge too strongly the service which would be done to the London hospitals were the system generally adopted. The *Express* perhaps hardly realises how much success would mean to the voluntary system. It may interest the Editor to know how difficult it has been hitherto to convince London hospital men of the practicability of an extended system of weekly contributions in London. We have seen energetic chairmen and hospital secretaries spare no effort to ensure the success of an appeal; and we have also seen a look of deep scepticism when the same men have been invited to attempt to get weekly contributions in London. It was almost as if they did not know how to set about the work. Sometimes, almost weary of our own advocacy, we have wondered if this reluctance sprang from the fact that the success of an appeal redounds to the personal satisfaction of the person who signs it. We have heard such people say, merely because their letter had been generously treated throughout the London newspapers: "£20,000 as the result of our letter is not bad," as if equal publicity would not have produced the same result over another name. All the machinery for getting money was there. The method was familiar; the effort very simple; the personal satisfaction very great.

To start, however, a system of weekly contributions in London, where, despite the work of the Metropolitan Hospital Saturday Fund, these collections are the exception, not the rule, demands somewhat tougher qualities. And it must be admitted that up to the present London hospital men have not displayed them. Whether inspired of themselves or by their chief, the employees of Messrs. W. H. Smith have set the example. They have begun the extension all desire; and, in view of what has been said above, it is remarkable that the scheme sprang from a firm, and not—at all events, directly—from any hospital authority. We have hopes, therefore, that the employees of other firms will be interested and wish to do similar service and win similar credit. If the impulse is given from staff to staff, and from the head of one firm to another, it can hardly fail of general adoption; nor, once the plan has started, will the most pessimistic London hospital secretary oppose it! Of that at least we may be sure. If the *Daily Express* will bear these facts in mind it will continue to encourage what is potentially a very large source of revenue. Nor can we show our appreciation better, we believe, than by informing our new ally of the difficulties which have hitherto beset the scheme. At all events, a newspaper effort to make the system general in London will do much greater service than was done by the scaremongers of a year and less ago.

